# Mendelian Randomization Identifies the Potential Causal Impact of Dietary Patterns on Circulating Blood Metabolites

**DOI:** 10.3389/fgene.2021.738265

**Published:** 2021-11-01

**Authors:** Nele Taba, Hanna-Kristel Valge, Andres Metspalu, Tõnu Esko, James F. Wilson, Krista Fischer, Nicola Pirastu

**Affiliations:** ^1^ Estonian Genome Centre, Institute of Genomics, University of Tartu, Tartu, Estonia; ^2^ Institute of Molecular and Cell Biology, University of Tartu, Tartu, Estonia; ^3^ Faculty of Medicine, University of Tartu, Tartu, Estonia; ^4^ Program in Medical and Population Genetics, Broad Institute, Cambridge, MA, United States; ^5^ Centre for Global Health Research, Usher Institute, University of Edinburgh, Edinburgh, United Kingdom; ^6^ MRC Human Genetics Unit, Western General Hospital, Institute of Genetics and Cancer, University of Edinburgh, Edinburgh, United Kingdom; ^7^ Institute of Mathematics and Statistics, University of Tartu, Tartu, Estonia

**Keywords:** dietary intake, nutrition, metabolomics, two-sample mendelian randomization, dietary patterns, lipid metabolism

## Abstract

Nutrition plays an important role in the development and progress of several health conditions, but the exact mechanism is often still unclear. Blood metabolites are likely candidates to be mediating these relationships, as their levels are strongly dependent on the frequency of consumption of several foods/drinks. Understanding the causal effect of food on metabolites is thus of extreme importance. To establish these effects, we utilized two-sample Mendelian randomization using the genetic variants associated with dietary traits as instrumental variables. The estimates of single-nucleotide polymorphisms’ effects on exposures were obtained from a recent genome-wide association study (GWAS) of 25 individual and 15 principal-component dietary traits, whereas the ones for outcomes were obtained from a GWAS of 123 blood metabolites measured by nuclear magnetic resonance spectroscopy. We identified 413 potentially causal links between food and metabolites, replicating previous findings, such as the association between increased oily fish consumption and higher DHA, and highlighting several novel associations. Most of the associations were related to very-low-density, intermediate-density (IDL), and low-density lipoproteins (LDL). For example, we found that constituents of IDL particles and large LDL particles were raised by coffee and alcohol while lowered by an overall healthier diet and fruit consumption. Our findings provide a strong base of evidence for planning future RCTs aimed at understanding the role of diet in determining blood metabolite levels.

## Introduction

Nutrition plays an important role in the development and progress of several diseases, such as obesity (Popkin, 2001), type II diabetes (T2D) (Schwingshackl et al., 2017), cardiovascular diseases (CVD) (Boeing et al., 2012; Dilis et al., 2012; Schaefer, 2002), and cancer (Key et al., 2004; Johnson and Lund, 2007). These in turn create a high burden for individuals, society, the economy, and healthcare, and thus prevention is of great importance. In many cases, the mechanism by which food consumption acts on health is still unclear. Blood metabolites are promising candidates for filling this gap. Metabolites have been shown to be important in the onset of a wide range of diseases such as type II diabetes (Suhre et al., 2010; Wang et al., 2011), incident cardiovascular events (Würtz et al., 2015; Holmes et al., 2018), dementia (Lee et al., 2018; Tynkkynen et al., 2018), and colorectal cancer (Guertin et al., 2015; Shu et al., 2018) and indicative of mortality (Fischer et al., 2014), and are thus likely mediators for at least some of the food–health relationships. Furthermore, metabolites can serve as objective biomarkers of dietary intake. However, it is important to note that a metabolite can be an intermediate in the diet–disease relationship while not being an objective biomarker—this occurs for example when a metabolite is affected by several exposures. On the other hand, a metabolite can be an objective biomarker of dietary intake while not acting as an intermediate in diet–disease relationships. The main focus of this paper is investigating the diet–metabolite associations where metabolites may act as intermediates in diet–disease relationships*.*

In recent years, the progress in quantifying metabolites has allowed investigation of the relationship between food and blood or urine metabolite levels. Studies have shown associations between metabolomic profile and intake of fruit and vegetables (Menni et al., 2013), coffee (Guertin et al., 2015), alcohol (Würtz et al., 2016), and a wide range of dietary patterns. A more detailed overview of the current state of the field is summarized by Guasch-Ferré, Bhupathiraju, and Hu (Guasch-Ferré et al., 2018) and Brennan and Hu (Brennan and Hu, 2019). Most studies in this field are observational and are thus limited by the typical biases which affect nutritional epidemiology (i.e., reporting bias, strong correlation between the studied variables, etc.) and are therefore unfit to detect causal relationships. Moreover, even in feeding studies conducted under very strict and controlled conditions, the effects could be measured only on a short-term basis and on limited sample size, which in turn limits statistical power.

Nevertheless, the feeding studies provide convincing evidence that dietary intake has causal effects on the metabolic profile, highlighting the potential to assess dietary intake *via* investigation of metabolomic profiles. Such studies have been applied to the percentage of dietary intake coming from carbohydrates/fat/protein (Esko et al., 2017), the effects of a low-glycemic index diet (Hernández-Alonso et al., 2019), and the dose-dependent effect of orange juice on proline betaine (Gibbons et al., 2017). Consequently, consumption or nonconsumption of various dietary items can cause change in the levels of blood metabolites, which are therefore likely candidates to be acting as intermediates between food and health. Thus, detecting potentially causal relationships between dietary choices and blood metabolites might reveal more insight into the mechanism by which food affects health.

A possible complementary approach to observational and feeding studies is the method of Mendelian randomization (MR) (Davey Smith and Ebrahim, 2003). MR exploits the natural randomization of the alleles associated with exposure in the population to measure the long-term effects of the trait on the outcome of interest. MR assumes that differences in exposure originating from different allelic compositions remain throughout life and, thus, by comparing the effect of the allele on the exposure and the outcome, it is possible to derive the effect of the exposure on the outcome.

MR relies on the results coming from genome-wide association studies (GWAS) which are generally publicly available and does not require the direct involvement of the participants. It is thus extremely cost-effective, and it is possible to apply it to contexts where randomized controlled trials would be unethical (for example, alcohol consumption). If carefully conducted, MR is exempt from the biases that are typical of observational studies. MR has been successfully used in many different contexts including nutritional epidemiology in the cases, for example, of milk (Bergholdt et al., 2015; Yang et al., 2017), alcohol (Chen et al., 2008; Andrews et al., 2020), and coffee consumption (Nordestgaard et al., 2015; Lee, 2018). Nevertheless, there are no studies using MR on a broader range of dietary items due to the lack of single-nucleotide polymorphisms (SNPs) strongly associated with food consumption to be used as instrumental variables.

By virtue of the availability of the data from UK Biobank, we have recently been able to broaden the number of foods for which genetic instruments are available, identifying several potentially causal food–health relationships (31, *currently available as preprint*). We thus decided to use MR to investigate the potentially causal effect of 40 foods/dietary patterns on 123 blood metabolites measured by nuclear magnetic resonance spectroscopy (NMR) available from a previous large GWAS by Kettunen et al. (2016). We detected 413 potentially causal links between food and metabolites, replicating previous findings and bringing novel insights, and discuss how these may be mediating the effect of food on health.

## Methods

**FIGURE 1:**
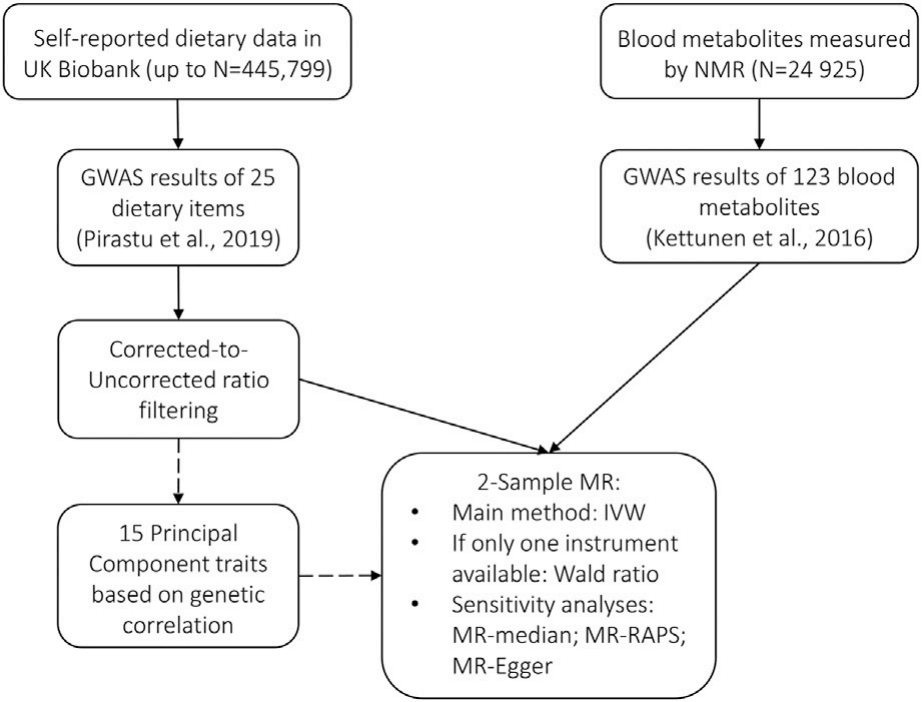
Selection of instrumental variables and workflow.

To infer the potentially causal relationships between food and metabolites, we used two-sample MR. In contrast to conventional MR, which would require instrument, exposure, and outcome to be available as individual-level data for the same samples, the same analysis can be performed using summary statistics from GWAS. In this case, instead of directly estimating the effect of the SNP on the exposure and on the outcome, the parameter estimates from previous association studies for the two variables are used. MR can thus be performed, even if sample sizes of the two studies are different and there is no sample overlap—in the latter case, the method is called two-sample MR (Pierce and Burgess, 2013). Apart from the obvious advantage of using the existing summary statistics, two-sample MR minimizes the potential residual genetic confounding. We performed two-sample MR using MR-Base *via* R-package TwoSampleMR version 0.5.5 (Hemani et al., 2018) (see detailed description: https://mrcieu.github.io/TwoSampleMR/). The selection process of instrumental variables and workflow is summarized in Figure 1.

### Dietary Traits and Principal Components

For our study, exposure instruments were selected from the previous GWAS on food consumption conducted in United Kingdom Biobank data (up to *N* = 445,799) (Pirastu et al., 2019) which included 25 traits for which valid instruments were available: consumption of beef, beer, bread, champagne/white wine, cheese, cooked vegetables, decaffeinated coffee, dried fruit, fresh fruit, ground coffee, instant coffee, lamb, nonoily fish, oily fish, pork, poultry, processed meat, red wine, salad, salt, spirits, tea, water adjusted for coffee, and vegetarianism and drink temperature. The paper reporting the results of food consumption GWAS is currently under review and has not been published yet and consequently included in the current study as a preprint. The replication analyses for the food consumption GWAS were conducted on two different cohorts: the EPIC-Norfolk Study and the Fenland Study. The statistical power of the replication cohorts was relatively limited (sample sizes 21,337 and 11,442 individuals, respectively). Despite that, for 82% of the signals a concordant direction of effects was observed and for 32% of the signals the nominal significance was achieved (Pirastu et al., 2019). The performance of the United Kingdom Biobank dietary questionnaire has been previously assessed and shown to reliably rank individuals according to the intake of the measured foods and food groups (Bradbury et al., 2018).

**FIGURE 2:**
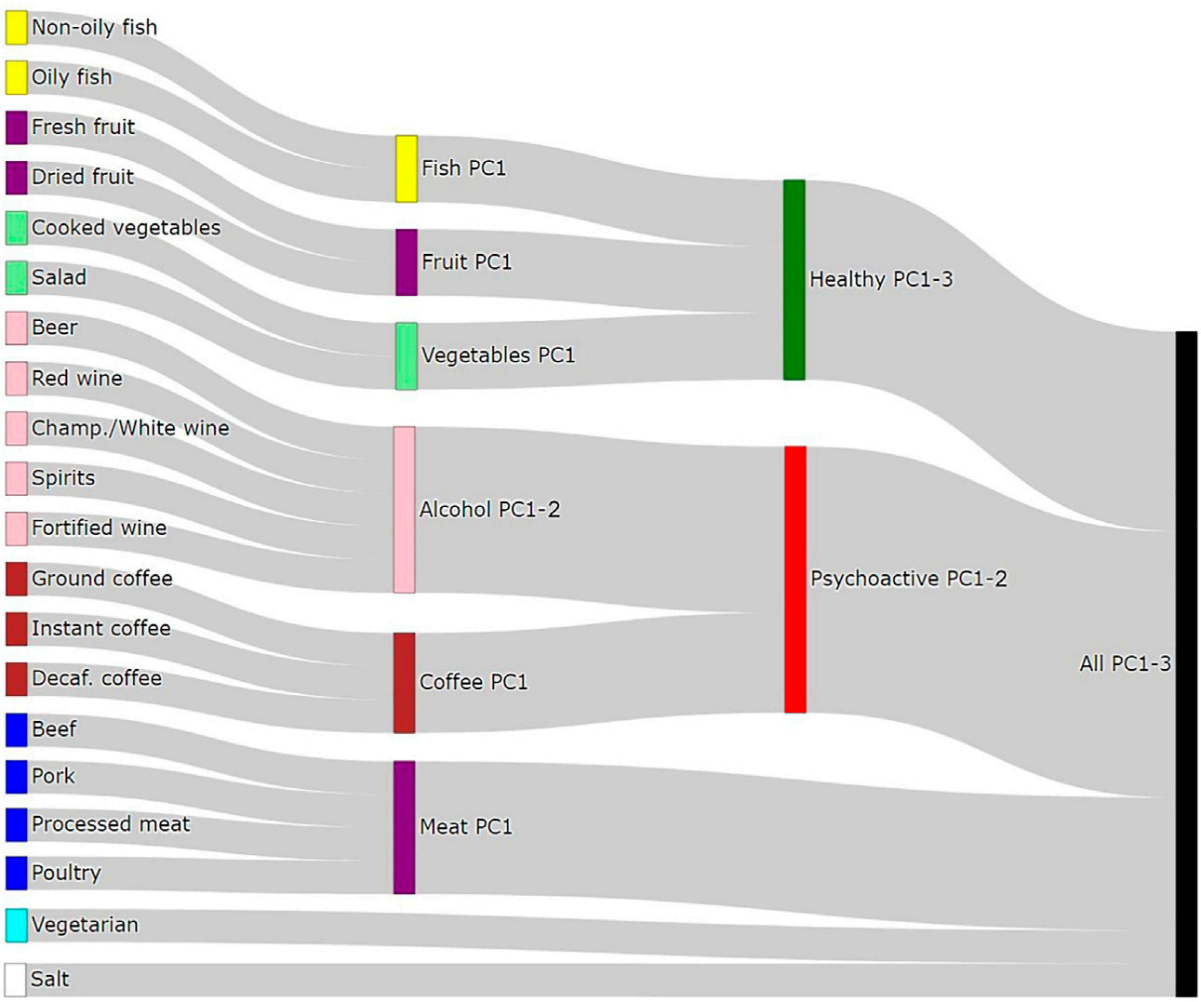
Sankey diagram of the relationships between dietary items and the principal components traits.

**FIGURE 3:**
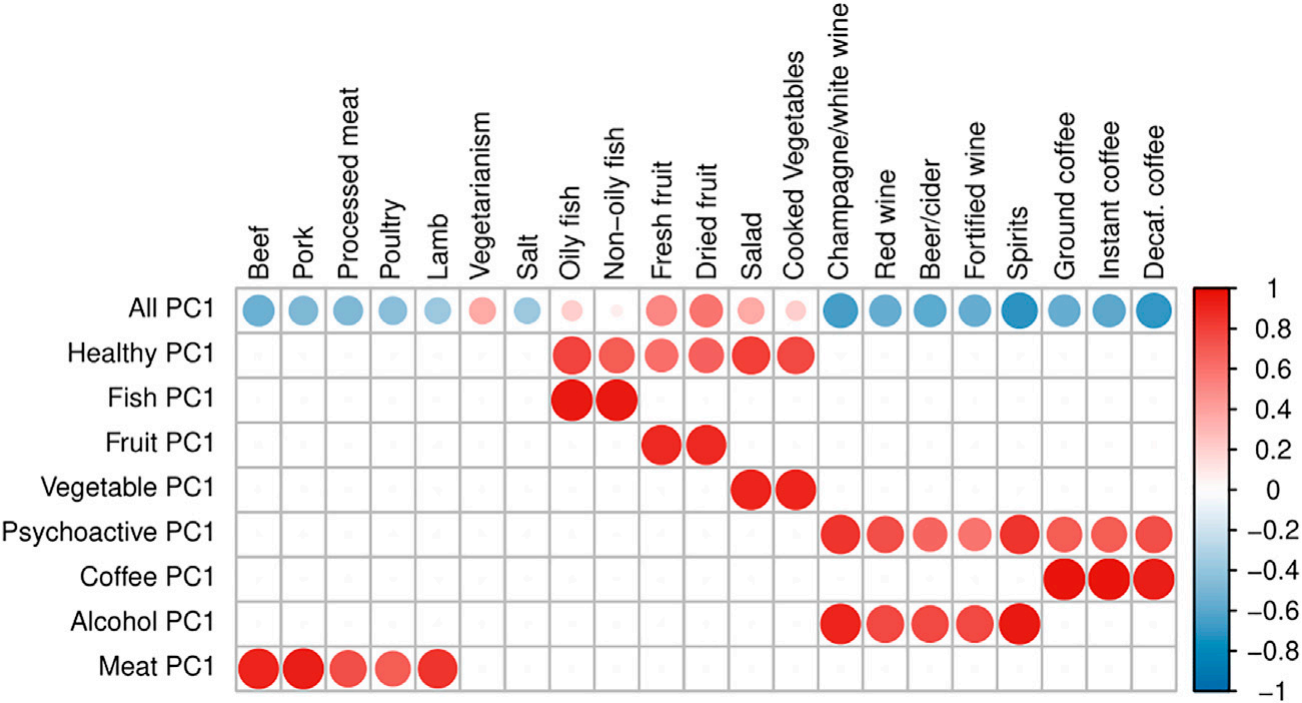
Loadings of PC traits. The plot represents the loadings of separate dietary items on each of the main PC-traits. Blank squares indicate that the corresponding item is not a component of the PC-trait. The size of the dots and the color intensity are proportional to the magnitude of the loadings, while the color indicates the sign of the loading: a darker red refers to a stronger positive loading, whereas darker blue corresponds to stronger negative loading.

In order to be able to distinguish between the independent effects of single food items and those arising due to the effect of dietary patterns, we defined 15 “Principal Component traits” (PC-traits) by first clustering the single food items using the iCLUST algorithm (Revelle, 1979; Revelle and Zinbarg, 2009). The defining and calculation of PC-traits is not a part of this paper and was done for our previous manuscript—therefore, only a brief description is included here and more detailed information can be found in Pirastu et al. (2019). After clustering the single-food items, we split the resulting tree dendrogram into different layers depending on the items in each cluster and the degree of similarity. For example, Oily-Fish and Non-Oily fish were first grouped in an overall fish consumption variable (Fish-PC1) and then in a more general healthy food measure together with Fruit-PC1 and Vegetables-PC1. Finally, they were all used to estimate a measure of the overall dietary pattern (All-PC1-3). Figure 2 shows the Sankey plot of the relationships between the different defined traits, and Figure 3 represents the loadings of each single food/drink on each of the main PC-traits (a full table of the PC-loadings can be viewed in Supplementary Table S1 and is visualized in Supplementary Figure S1). Higher values of All-PC1 correspond to higher consumption of vegetables, fruit, and fish and lower consumption of meat, coffee, and alcohol. All-PC2 separates foods from drinks (alcohol-containing beverages and coffee) with higher values corresponding to higher consumption of coffee and alcohol and lower consumption of the rest of the foods. Fruit-PC1 corresponds to higher consumption of dried and fresh fruit whereas a higher value on Psychoactive-PC1 corresponds to higher consumption of coffee and alcohol.

The SNP effect estimates for the PC-traits were estimated by applying principal component analysis to the genetic correlation matrix of the items included in each group and by using the resulting rotation matrix to project the estimated effects of the SNPs on each of the items onto the PC space. Standard errors for the PC-trait effects were estimated using the single items SEs and the phenotypic correlation matrix.

### Instrumental Variable SNP Selection

As instruments, independent (r2<0.001) SNPs significantly associated with the traits in the exposure GWAS (*p*-value < 5 × 10–8) were used, for which the effect was not mediated through other confounders or health-related traits. In case of the PC-traits, SNPs were selected if they were associated (*p*-value <5e-8) with at least one of the items which participated in the trait definitions. Once extracted, each selected SNP was assigned the *p*-value among the traits of interest from the rotated PC space. Next, the SNPs that were not available in the outcome GWAS were excluded. Finally, we applied LD pruning (r < 0.001) to the selected SNPs. We have previously shown that food frequency GWAS results are strongly affected by educational attainment (as a proxy of socioeconomic status) and by health-related traits such as body mass index, blood pressure, and cholesterol, for which dietary advice is generally given (Pirastu et al., 2019). This results in either bias by indication (where the behavior is determined by health advice or belief, e.g., lower fat consumption in people with high cholesterol) or reporting bias (where people underreport or overreport food consumption due to their health status, e.g., obese people underreport true fat consumption). If the biasing trait is heritable, this leads to spurious results in the GWAS, which can bias the MR results. In order to distinguish which variables are likely directly associated with the food of interest (and not mediated by health conditions), we have previously developed a method called Corrected to Uncorrected ratio filtering (CUR), which is based on the idea that if the SNP is directly associated with food preferences, then its effect should not change when adjusted for education status or health conditions (Pirastu et al., 2019). The following list was considered as potential confounders: body mass index, low-density lipoprotein cholesterol, high-density lipoprotein cholesterol, triglycerides, diastolic and systolic blood pressure, T2D, coronary artery disease, Crohn’s disease, ulcerative colitis, and educational attainment. Briefly, the method is composed of three steps. The first step is to conduct multivariable MR using each food trait as the outcome while the exposures are selected through a stepwise procedure. Once the effect of each exposure is obtained for each SNP, we obtain an “expected effect” which represents the overall effect of each SNP on the food trait mediated through the causal exposures. We then obtain a corrected effect which is the difference between the observed effect and the exposure-mediated effect. Finally, we estimate the ratio between the corrected effect the uncorrected observed effect (CUR). When CUR is close to 1, it means that the effect of the SNP on the food trait is entirely direct and thus it is a valid instrument. We have shown elsewhere (Pirastu et al., 2019) that using SNPs with CUR = 1 ± 0.05 maximizes the chances of selecting the correct SNPs. The strength of the instruments was assessed by using F-statistic and is reported in Supplementary Table S2. The SNP effects for the outcomes were obtained from a previous GWAS on 123 plasma metabolites in 24,925 individuals (Kettunen et al., 2016). Most of the metabolites assessed in this GWAS were linked to the lipid profile, and consequently the sample of metabolites used in the current paper is biased toward lipids.

### Mendelian Randomization Methods and Sensitivity Analysis

As the main MR method, we used the inverse-variance weighted method, with a random-effect standard error if the heterogeneity *p*-value was less than 0.05/123. One of the problems of MR is when SNPs are associated with the outcome through causal paths which do not pass through the exposure of interest, also referred to as horizontal pleiotropy. In order to remove the SNPs with the highest heterogeneity (and thus likely are subject to horizontal pleiotropy), we used the method called MR-Radial (Bowden et al., 2018). All analyses were thus run on the instruments selected with this method. As sensitivity analyses, we used MR-Median (Bowden et al., 2016), MR-RAPS (Zhao et al., 2019), and MR-Egger (Bowden et al., 2015). These methods have been thoroughly described elsewhere and are all sensitive to the breaking of different MR assumptions. When only one instrument was available, the Wald ratio method was used. Finally, we defined as significant the food–metabolite relationships where Storey’s q-value (Storey, 2003) was less than 0.05. The analyses were run using R version 3.6.1 (R Core Team, 2019).

## Results

**FIGURE 4:**
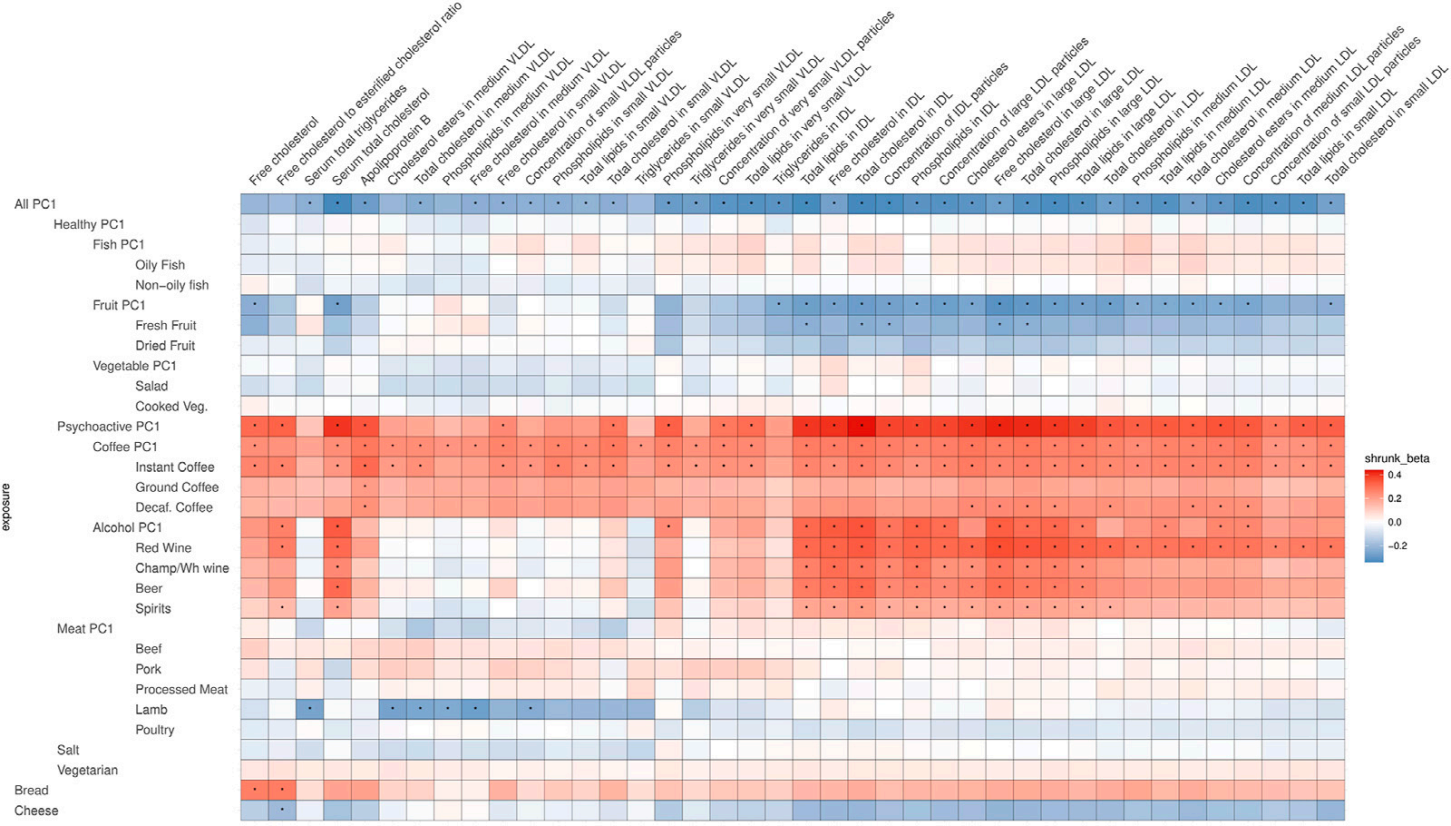
Heatmap of the relations of food traits with atherogenic lipoproteins and related metabolites (VLDL, IDL, LDL, and related). Depicted are only the metabolites which showed a significant association with the food items or groups. To facilitate meaningful visualization and maximize the appearance of signal rather than noise, we applied a shrinkage method—imposing a Bayesian prior assumption on the distribution of beta (mean 0, SD 0.1), and conjugating that with the likelihood of our results and then taking the mean beta from the resulting distribution, thus shrinking estimates with larger SEs more toward 0. The color of the squares indicates the size and direction of betas after a shrinking procedure. The FDR-significant results are marked with “*” in the middle of the square.

**FIGURE 5:**
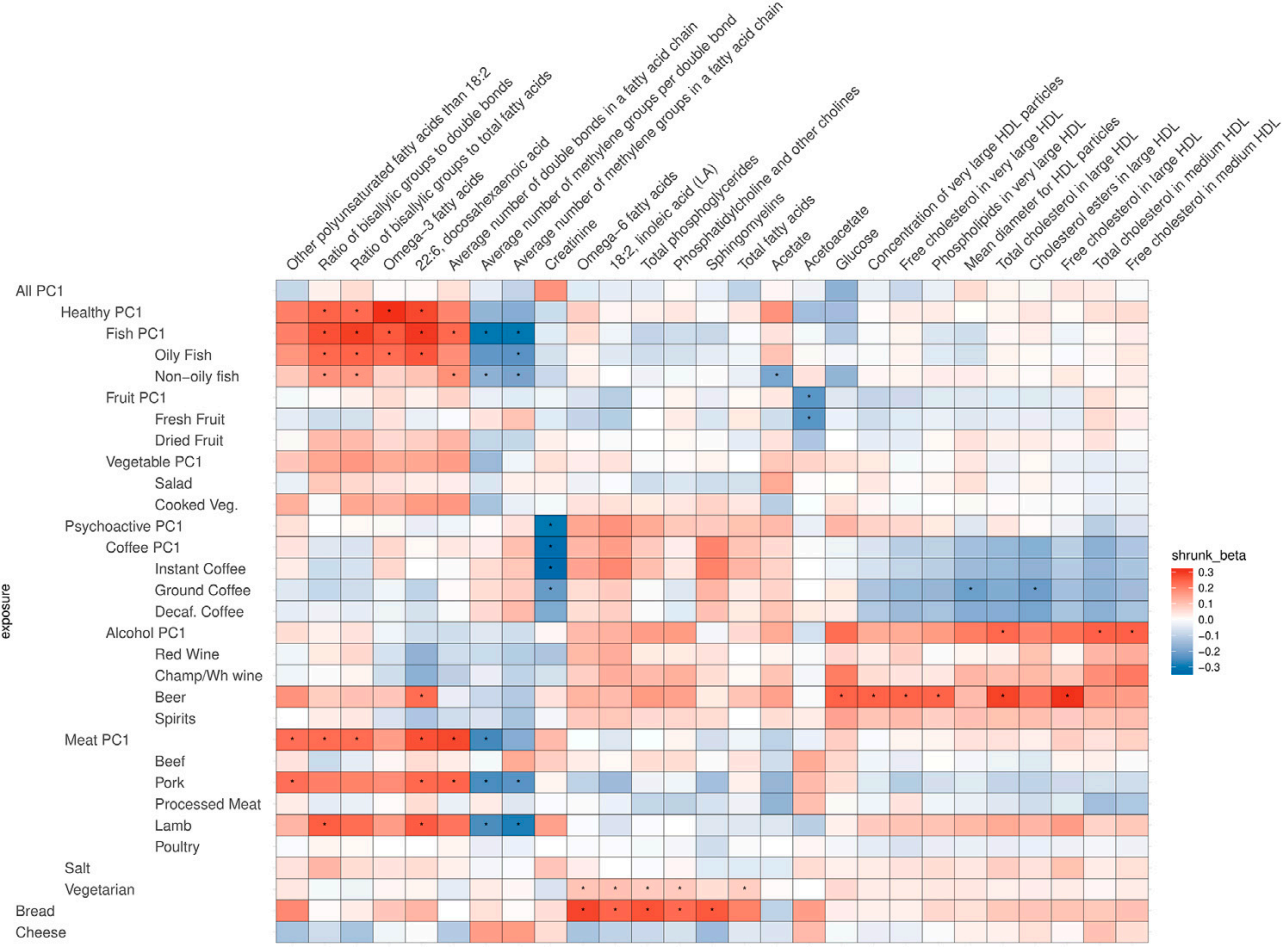
Heatmap of the relations of food traits and all other metabolites (not VLDL, IDL, LDL related). Depicted are only the metabolites which showed a significant association with the food items or groups. To facilitate meaningful visualization and maximize the appearance of signal rather than noise, we applied a shrinkage method—imposing a Bayesian prior assumption on the distribution of beta (mean 0, SD 0.1), and conjugating that with the likelihood of our results and then taking the mean beta from the resulting distribution, thus shrinking estimates with larger SEs more toward 0. The color of the squares indicates the size and direction of betas after a shrinking procedure. The FDR-significant results are marked with “*” in the middle of the square.

After correcting for multiple testing by using the false discovery rate (FDR<0.05) *via* Storey’s q-values, 413 potentially causal relationships remained statistically significant. Most of these are associations related to atherogenic lipoproteins: very-low-density lipoproteins (VLDL), intermediate-density lipoproteins (IDL), and low-density lipoproteins (LDL), which all contain Apolipoprotein B (ApoB). Figures 4, 5 are heatmaps reporting the relationships between food items and groups with the metabolites, Figure 4 depicts atherogenic lipoproteins and related metabolites, and Figure 5 depicts all other food–metabolite relationships of interest. For readability, some metabolites which had only single significant associations are left out of these graphs. A full list of all significant food–metabolite relationships that we detected is found in Supplementary Table S3, and a full list of all food–metabolite relationships we analyzed is found in Supplementary Table S4.

### Sensitivity Analysis

Out of the 413 significant results, 19 were based on the Wald ratio and 394 were based on the inverse-variance weighted method. For the latter, we used MR-Median, MR-RAPS, and MR-Egger as sensitivity analyses to detect potential violation of assumptions. We found that the sensitivity analyses broadly agreed with the results of the main analysis with the exception of one case (Alcohol-PC2 on “Description of average fatty acid chain length, not actual carbon number”), where the direction of the effect estimate from MR-Median was opposite to the one from the main analysis, and thus this result cannot be considered reliable. In 16 cases (mostly when Alcohol-PC2 was the exposure), the betas from MR-median were smaller (>50% effect difference) than the ones in the main analysis, which indicates that in these cases the effect sizes might be inflated. For some food–metabolite pairs, we detected some indication of heterogeneity. This was mostly related to PC-traits which reflect the effects of many different exposures, and it is thus not unexpected.

### All-PC1

Most significant relationships were found with All-PC1 (higher value corresponds to lower meat/coffee/alcohol/salt and higher fruit/salad consumption), higher consumption of coffee (Coffee-PC1), and consumption of alcoholic beverages and coffee (Psychoactive-PC1 and -PC2). The majority of the effects of these traits were on the same metabolites (VLDL, IDL, and LDL related) and with opposite directions: namely, showing negative correlations for All-PC1 and positive correlations for Coffee-PC1 and Psychoactive-PC1. These effects were very similar to each other in size, with betas ranging from −0.502 to −0.322 for All-PC1, from 0.327 to 0.436 for Coffee-PC1, and from 0.425 to 0.778 for Psychoactive-PC1. The latter is likely partly due to the strong correlation between these lipid measurements.

All-PC1 had significant associations with 41 of the measured 123 metabolites, which was, as expected, the highest number of associations we saw for any of the tested foods or PCs. Interestingly, these effects were mostly related to atherogenic lipoproteins—the components of VLDL, IDL, and LDL of various sizes, and ApoB—whereas surprisingly there were no notable effects on any of the components of high-density lipoprotein (HDL) particles or other metabolites measured (for example, omega-3 or omega-6 fatty acids). Additionally, All-PC1 had significant effects on serum total cholesterol and serum total triglycerides. When a significant potentially causal relationship was found for a metabolite, the associations of this metabolite with each dietary item were examined. Often, the effects on PC-traits were driven only by a subset of dietary items. For example, in the case of total lipids in IDL, although we see a clear effect of overall diet (All-PC1), it seems that this is driven primarily by the components of Fruit-PC1 and Psychoactive-PC1 while the remaining foods do not seem to play any role. This observation may explain some of the significant heterogeneity we detected.

### Psychoactive-PC1, Alcohol-PC1, and Coffee-PC1

Some strikingly clear patterns can be noted, when examining the results that are depicted on Figure 4—for example, looking at the significant positive effects that Psychoactive-PC1 has on the components of IDL particles, large LDL particles, and serum total cholesterol. In the case of these results, both subcategories—coffee and alcohol—clearly contribute to the overall effect of Psychoactive-PC1. More importantly, all the four subcategories of alcohol show independently significant effects. All the significant effects that Psychoactive-PC1 has on the components of IDL particles and large LDL particles are accompanied by a contrary effect from Fruit-PC1, which shows significant negative effects. The latter indicates that higher consumption of fruits has a lowering effect on several IDL and large LDL components. In these cases, the effects of Fruit-PC1 are with the same direction as the effects of All-PC1 (“low meat/coffee/alcohol/salt + high fruit/salad”). Furthermore, this association pattern is also partly followed by the components of medium LDL particles, but in this case, when looking into the components of Psychoactive-PC1, alcohol seems to play a less important role compared to coffee.

In most of the cases, alcohol and coffee both contributed to the overall effect of Psychoactive-PC1, and we cannot distinguish between the effects of the components (although alcohol seems to play a slightly larger role in the case of IDL particles). Therefore, in most of the cases Alcohol-PC1, Coffee-PC1, and the items comprising these behave in the same way. Nevertheless, there is a group of clear and notable counterexamples: the components of medium VLDL and small VLDL particles. In these cases, Coffee-PC1 shows clear positive effects and all the betas of all the coffee subgroups agree with the direction of these effects, whereas Alcohol-PC1 and all of its subgroups show no clear effects and do not seem to be associated with the components of medium and small VLDL particles. Another interesting example that behaves differently from other items in the group of Psychoactive-PC1 is beer, which has significant positive effects on glucose (β = 0.43, 95% CI: 0.15; 0.70), concentration of very large HDL particles (β = 0.47, 95% CI: 0.16; 0.77), free cholesterol in very large HDL (β = 0.45, 95% CI: 0.16; 0.75), phospholipids in very large HDL (β = 0.46, 95% CI: 0.16; 0.76), total cholesterol in large HDL (β = 0.53, 95% CI: 0.23; 0.83), free cholesterol in large HDL (β = 0.57, 95% CI: 0.27; 0.86), and 22:6 docosahexaenoic acid (DHA, a subgroup of omega-3 fatty acids; β = 0.84, 95% CI: 0.32; 1.37). The latter is an example, where beer is clearly the odd-one-out compared to the effects of other alcohol subgroups, indicating that the effect comes from other ingredients in beer rather than alcohol.

### Meat-PC1

The effects of Meat-PC1 do not have much contribution from beef, processed meat, and poultry and are driven mostly by pork (in the case of polyunsaturated fatty acids than other 18:2, and average number of double bonds in a fatty acid chain) or lamb (in the case of ratio of bisallylic groups to total fatty acids) or both (ratio of bisallylic groups to double bonds, DHA, and average number of methylene groups per double bond). Furthermore, it is noteworthy that lamb as a separate item had significant negative effects on several components of medium, large, very large, and largest VLDL particles, namely, on total cholesterol, cholesterol esters, free cholesterol, and phospholipids in medium and large VLDL; total lipids and triglycerides in very large and largest VLDL; phospholipids in very large VLDL; concentration of small and large VLDL particles; and mean diameter for VLDL particles. Thus, lamb has significant negative effects on the components of those lipoproteins that are largest and with lowest density, whereas showing no notable effects on the components of any of the lipoproteins that are smaller and with higher density than small VLDL particles.

### Fish-PC1, Vegetarianism, and Bread

As expected, Fish-PC1 had significant effects on omega-3 fatty acids and DHA, and these effects were clearly driven by oily fish. Furthermore, looking at Figure 5, the results regarding creatinine notably stand out, namely, there is a lowering effect of Psychoactive-PC1 on creatinine, which is clearly driven by coffee (which in turn shows significant negative effect on creatinine). Surprisingly, vegetarianism and bread share largely the structure of effects—they both have significant effects on omega-6 fatty acids (β = 3.73, 95% CI: 1.75; 5.70; β = 0.67 95% CI: 0.30; 1.04, respectively), 18:2 linoleic acid (LA, a subgroup of omega-6 fatty acids; β = 3.83, 95% CI: 1.86; 5.80; β = 0.55 95% CI: 0.18; 0.91), phosphatidylcholine and other cholines (β = 3.27, 95% CI: 1.31; 5.24; β = 0.53 95% CI: 0.17; 0.89), and total phosphoglycerides (β = 3.19, 95% CI: 1.23; 5.16; β = 0.63 95% CI: 0.25; 1.00). Of note, the results of vegetarianism are based only on one instrument and the method of the Wald ratio was used.

## Discussion

In this study, we have assessed the effect of long-term exposure to single foods and food groups on blood metabolite profiles using Mendelian randomization. We have in general found that in many cases these changes are not due to specific food items but are related to general dietary patterns. This could be due to the fact that most of the metabolites assayed are linked to lipid profile and it does not exclude more specific biomarkers being discovered for single items.

Many of the results that we found elaborate the lipid profiles of lipoprotein subclasses in more detail than the previous studies. Our results regarding the effects of alcohol on IDL and large LDL-related lipids conflicted with some observational studies’ findings (Würtz et al., 2016; Du et al., 2020), but agreed with another MR-study (Rosoff et al., 2019), whereas in our study we showed that the same relationships hold for each of the alcohol subgroups as well. Our finding of vegetarianism raising 18:2 linoleic acid replicated a previous finding from a randomized trial conducted on subjects with T2D (Kahleova et al., 2013), whereas our results show that this finding applies for the general population in a larger sample as well. Furthermore, the finding of vegetarianism raising omega-6 replicated a previous observational finding (Kornsteiner et al., 2008); however, our MR-analysis showed the potential for a causal relationship. We also saw a significant positive effect of oily fish on omega-3 fatty acids and DHA, which are well-known causal relationships (Horrocks and Yeo, 1999). The fact that our results conflicted with some observational findings, but aligned with known causal relationships and with a randomized trial, highlights the strength of MR studies as an intermediate step between observational studies and clinical trials and validates the utility of our approach.

### Association Pattern on VLDL, LDL, and IDL

Many of our significant results were related to All-PC1 (“low meat/coffee/alcohol/salt + high fruit/salad”), which seems to affect mostly LDL, VLDL, IDL, and related subclasses. The fact that the effect sizes were very similar can be partly explained by the strong correlations between the different metabolites explained in the Results section. Another explanation might be the effect that All-PC1 has on ApoB. Namely, higher values on All-PC1 (corresponding to lower meat/coffee/alcohol/salt and higher fruit/salad) have a lowering effect on ApoB. The latter is the protein part of LDL, IDL, and VLDL, specifically one ApoB per each lipoprotein, thus reflecting the total amount of atherogenic lipoprotein particles (Elovson et al., 1988). This is important in the context of health risks, because it shows that ApoB, being a component of all atherogenic lipoprotein particles, might reflect the actual CVD risk better than the amount of cholesterol in any lipoprotein particle type on their own (Leslie, 2017). Ference et al. (2019) came to the conclusion that the risk of CVD posed by LDL particles is more determined by the concentration of LDL particles measured by ApoB compared to the mass of cholesterol or triglycerides in LDL particles. Furthermore, a recent study using multivariable MR found that ApoB has a robust elevating effect on CVD risk, while the effects of LDL-C, triglycerides, and HDL-C on CVD risk were not significant after accounting for ApoB (Richardson et al., 2020). Our results confirm the possible role of ApoB as a substantial factor in the relationship between diet and health and thus can prove useful to investigate when the causal effects of dietary patterns on risk of CVD are of interest. Nevertheless, despite the important role of ApoB, there is still a wide body of literature showing other features of atherogenic lipoproteins affecting health risks and these should not be neglected when interpreting the findings.

One of the most notable patterns in our results was the one regarding IDL and LDL particles, where higher consumption of coffee and alcohol had elevating effects, whereas higher consumption of fruits and higher value on All-PC1 had lowering effects. Higher levels of atherogenic particles and their components is part of a less desirable blood lipoprotein profile due to the elevated risk for several cardiovascular diseases (Carmena et al., 2004; Ference et al., 2017). For example, IDL-C has been shown to be associated with the degree and frequency of CAD independent of LDL-C (Tatami et al., 1981). Furthermore, different properties of LDL, like size, amount of cholesterol esters and cholesterol, and fatty acid composition are all considered to be aspects of its CVD-causing capability (Lada and Rudel, 2004). The clear pattern of effects we saw on IDL and LDL indicates that these metabolites are likely affected by dietary habits. Since these metabolites are largely shown to affect cardiovascular health, and Mendelian randomization results indicate possible causal pathways, there is considerable scope for further investigation of these results. Furthermore, our results indicate that the harmful effects alcohol and coffee have on cardiovascular health are at least partly mediated by IDL and LDL lipoproteins.

### Possible Beneficial Effects of Alcoholic Beverages on Lipid Profile

There was a surprising positive effect of beer on DHA, which has been shown to have a cardioprotective effect, as concluded in a recent large meta-analysis of randomized control trials (Bernasconi et al., 2020). This effect on DHA was in the opposite direction compared to other alcoholic beverages, indicating that the beneficial effect is likely not due to alcohol itself but some other ingredient in beer. We propose that the beer–DHA relationship is worth further investigation, and it would be especially interesting to compare regular beer with nonalcoholic beer to detect whether the potential beneficial effect remains. Furthermore, even if beer had some beneficial effect, this is unlikely to counterbalance the negative effect of alcohol raising IDL and LDL and their very well-established effect in predisposing to cardiovascular disease.

Many observational and epidemiological studies have pointed to the possibility of moderate alcohol intake to have a protective effect from cardiovascular diseases (Ronksley et al., 2011) and beneficial effects on cardiovascular biomarkers (Brien et al., 2011). The positive effects of alcohol on the lipid profile have mostly been associated with raised HDL-C levels (Brien et al., 2011). Although our results do show such a trend (with only beer having a significant effect on HDL levels), there have been conflicting results in other studies on the actual benefit of higher HDL-C levels protecting from developing cardiovascular diseases (Briel et al., 2009). Looking at the possible beneficial effect from raised HDL, one cannot underestimate the negative impact of raised IDL and LDL levels on health due to alcohol consumption. Furthermore, the reduced risk of cardiovascular disease in the case of light and moderate alcohol consumption compared with abstinence and heavy drinking, or in other words the U-shaped association of alcohol and CVD risk, found in many epidemiological studies has been found to be originating from other factors such as abstaining from alcohol due to poor health (Shaper et al., 1988) or might be resulting from reverse causation (Millwood et al., 2019). Further, alcohol has been shown to have harmful effects on health regardless of the quantities consumed (Holmes et al., 2014). Thus, the beneficial effects of alcohol on health are debatable and our results do not indicate that alcohol itself would have any beneficial effects.

### Coffee and Lipid Profile

We saw a significant increasing effect from Coffee-PC1 on several VLDL, IDL, and LDL lipoprotein subclasses and their components and in addition a negative effect on the mean HDL diameter and cholesterol esters in large HDL. Effects on other HDL parameters were not significant, but there is a notable trend toward a negative correlation. Overall, a higher value on Coffee-PC1 results in a more unhealthy lipid profile, raising ApoB, serum total cholesterol, VLDL, IDL, and LDL levels, and their constituents. There is consistent evidence in the literature of coffee raising LDL-C and total cholesterol. Poole et al. (2017) showed in their meta-analysis of randomized clinical trials (RCTs) that coffee has elevating effects on total and LDL cholesterol, but no clear effect on HDL cholesterol. Furthermore, coffee consumption has been associated with elevated ApoB levels (Williams et al., 1985; Periti et al., 1990; Cornelis and van Dam, 2020), but the causal link has not been established. Interestingly, Cornelis and van Dam (2020) observed higher HDL-C associated with higher coffee consumption, which is not in line with previous RCTs showing no significant effect and contrary to our results showing a non-significant HDL-C lowering effect. Thus, the effect of coffee on HDL-C and other HDL-related metabolites remains unclear.

We are not aware of any studies showing the effects of coffee on IDL and VLDL particles or their constituents, for which we observed consistent elevating effects. Raised VLDL levels might be due to cafestol, a common ingredient in coffee, having an effect of increased VLDL particle assembly rate on the liver (de Roos et al., 2001). Furthermore, we are the first ones to observe the effect of coffee on such a wide range of lipoprotein subclasses, allowing us to see the consistency of coffee’s effects on atherogenic lipoproteins. Looking at these results in the context of health outcomes, there is considerable scope for further investigation. In the case of coffee, the results regarding cardiovascular health are controversial and mostly show either beneficial or neutral effects (Chrysant, 2015) or that moderate consumption is unlikely to have adverse effects (Rebello and van Dam, 2013). On the other hand, raised ApoB levels indicate that the overall atherogenic particle amount is higher, which, as mentioned before, is a good predictor of CVD risk. Overall, we did not see any beneficial effect of coffee on lipid profile in our results; in fact, coffee turned out to have a negative effect on a larger variety of atherogenic lipoproteins than other dietary items. Hence, the total effect of coffee on health remains still unclear, but we propose that the potential harmful effect of coffee on health might be mediated by ApoB and thus *via* VLDL, IDL, and LDL. Furthermore, these results suggest that coffee consumption should be limited in people at risk of cardiovascular diseases.

### Study Limitations and Strengths

Our study has several limitations: for some items, only a few SNPs were available to be used as an instrument in MR; self-reported dietary data are a difficult trait to investigate since it encompasses several biases—we aimed to mitigate this issue by using the corrected-to-uncorrected ratio (Pirastu et al., 2019); MR analysis can suffer from horizontal pleiotropy—we tried to mitigate this issue by using sensitivity analyses; item heterogeneity (the same dietary trait can incorporate items with varying nutritional values); sample homogeneity whereby our analyses are conducted on the European population and might not be fully generalizable to the population of the whole world; and when analyzing dietary patterns, we are limited to the ones arising from the data and cannot therefore fully account for the context of different diets. Furthermore, despite using the corrected-to-uncorrected ratio to identify SNPs that are not influenced by confounders, we cannot rule out the possibility that there were additional confounders that were not included in the model or that some might not have been captured properly (for example, when using educational attainment as a proxy for socioeconomic status). Nevertheless, our study has several strengths: to our best knowledge, we are the first ones to perform MR analysis between dietary items and blood metabolites with such a large amount of dietary SNP instruments available; we found several distinct patterns that shed more light on how dietary changes might affect cardiovascular health; and we found multiple interesting associations that are worth further investigation *via* feeding studies or randomized trials. We investigated the effects on metabolites profiled with NMR spectroscopy, which encompass mostly lipid profiles. Future studies with proteomics and metabolite data from mass spectrometry might give more detailed insight about the mechanisms by which food affects health.

## Conclusion

In conclusion, we aimed to investigate the relationships between dietary items and blood metabolites in order to gain more insight into the mechanisms by which food affects health. Mendelian randomization proved a useful method for fulfilling this aim. Moreover, we replicated several previous findings and known associations, which validates the method used. We did not detect any reported causal relationships in the literature conflicting with our results; however, occasionally our results conflicted with previous observational studies. This demonstrates the strength of MR studies and indicates that some of the previously reported findings might be confounded through other unobserved factors. Nevertheless, in order to give actual dietary intervention suggestions, additional thorough investigations should be carried out *via* feeding studies or randomized trials. We believe that many of the potentially causal relationships that have been described here have promising potential for further investigation.

## Data Availability

Publicly available datasets were analyzed in this study. This data can be found here: All harmonized data for the exposure GWAS are within supplementary files. Outcome GWAS can be accessed here: https://www.ebi.ac.uk/gwas/publications/27005778.

## Supplementary Material

Supplementary Figure S1 Loadings of all principal component traits. The plot represents the loadings of separate 482 dietary items on each of the PC-traits. Blank squares indicate that the corresponding item is not a 483 component of the PC-trait. The size of the dots corresponds to the magnitude of the loadings whereas 484 the color of the dots describe in addition the direction of the loadings.
