# Supplementary figures and images for: Mendelian Randomization Identifies the Potential Causal Impact of Dietary Patterns on Circulating Blood Metabolites
Source: Front Genet. 2021 Nov 1;12:738265. doi: 10.3389/fgene.2021.738265 (PMC8592281; doi:10.3389/fgene.2021.738265)

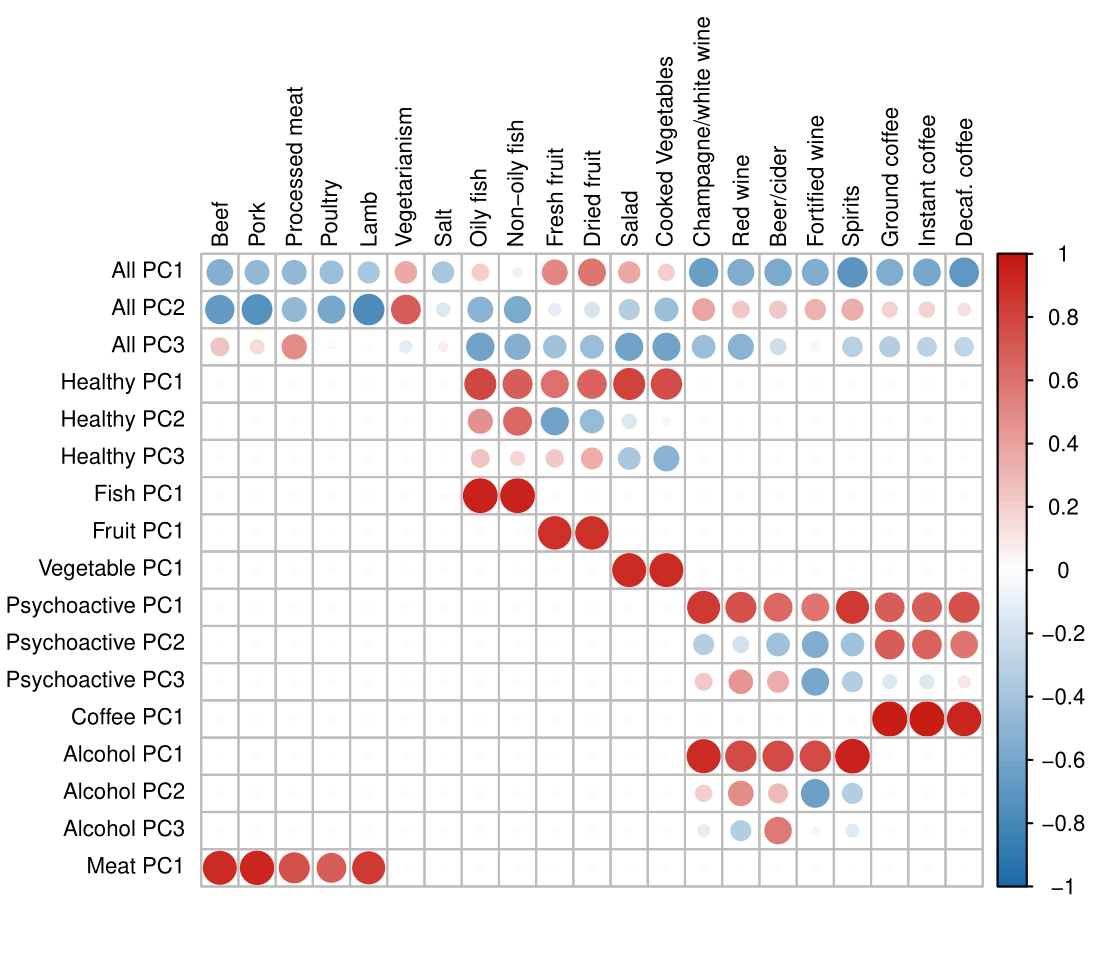

Supplement: Supplementary file 1 [file Image1.TIFF]
